# Hepatitis C Virus-Infected Responders and Relapsers to Treatment Show Similar Genetic Profiles of* IL28B* and* IL10* Single Nucleotide Polymorphisms

**DOI:** 10.1155/2018/2931486

**Published:** 2018-05-20

**Authors:** Suiane Lima de Souza, Luãnna Liebscher Vidal, Juliene Ramos, Cynthia Chester Cardoso, Henrique Sérgio Moraes Coelho, Cristiane Alves Villela-Nogueira, Renata de Mello Perez, Marcelo Alves Soares, André Felipe Santos

**Affiliations:** ^1^Universidade Federal do Rio de Janeiro, Rio de Janeiro, RJ, Brazil; ^2^D'Or Institute for Research and Education (IDOR), Rio de Janeiro, RJ, Brazil; ^3^Instituto Nacional do Câncer, Rio de Janeiro, RJ, Brazil

## Abstract

Genotype 1 of hepatitis C virus (HCV) is the most prevalent worldwide. Pegylated-interferon and ribavirin therapy is still used in the developing world but has less efficiency in this genotype. Single nucleotide polymorphisms (SNPs) rs12979860 and rs8099917 (IL28B) and rs1800896, rs1800871, and rs1800872 (IL10) are related to treatment outcome, but previous studies clustered nonresponse and relapse patients. The aim of this study is to analyze the frequency of those SNPs in HCV genotype 1 for response, nonresponse, or relapse. Patients were classified according to treatment outcome. Genomic DNA was extracted by blood samples and SNPs were defined by PCR and sequencing. Data analysis was performed with R project. The frequency of rs12979860 CC was similar among responders (0.48) and relapsers (0.46) and lower among nonresponders (0.18). The same trend was observed for rs8099917 TT. rs12979860 CC showed a protective effect for relapsers compared to nonresponders (OR = 0.25) as it occurs with responders (OR = 0.17). Haplotypes 12979860/C rs8099917/T were associated with protection against the nonresponder phenotype compared to responders (OR = 0.27) or relapsers (OR = 0.37). Frequency of rs12979860 and rs8099917 is different between relapsers and nonresponders, but similar between relapsers and responders.

## 1. Introduction

Currently 130–150 million people, approximately 2% of the world population, are infected with the hepatitis C virus (HCV) worldwide [1]. HCV has seven acknowledged genotypes (1–7), but genotype 1 (gen1) is the most prevalent, being responsible for 46% of cases [2]. Despite such high prevalence, only 50% of HCV gen1-infected patients achieve sustained virological response (SVR) with pegylated-interferon (pegIFN) and ribavirin (RBV) dual therapy, while SVR occurs in 80% of patients with other genotypes [3]. Although this therapeutic regimen has been overcome by the development of direct acting antivirals (DAA), it is still widely used in the developing world.

The outcomes related to failure to pegIFN/RBV treatment are classified into nonresponse, when the HCV viral load does not decrease or decreases less than 2 log of the baseline HCV-RNA load, or relapse, when the viral load becomes undetectable at the end of treatment, but it is again detectable 24 weeks after the end of treatment [4]. Independent GWAS studies showed two single nucleotide polymorphisms (SNPs) close to the* IL28B* gene, rs12979860 C/T and rs8099917 T/G, which are related to the outcome of this treatment [5, 6].* IL10* SNPs rs1800896 A/G, rs1800871 C/T, and rs1800872 A/C, located, respectively, at positions -1082, -819, and -592 of the promoter region of that gene, are also related to pegIFN/RBV treatment outcome, but there is a controversial relationship between those SNPs and HCV. Some studies showed an association with treatment [7, 8], but others have shown no difference between failure patients and patients who achieve SVR [9, 10].

The previously mentioned studies that suggested the association of* IL28B* and* IL10* SNPs with SVR after pegIFN and RBV therapy clustered nonresponder and relapser patients in treatment failure. A multicenter study suggested different genetic frequencies of rs12979860 and rs8099917 between these two groups of patients [11] specifically for HCV gen2 and 3, and the analysis did not compare individually the nonresponders and the relapsers with the RVS patients. A detailed analysis of those types of patients in an individual manner, particularly in the context of HCV gen1 infection, is still lacking, what has prompted us to study the frequency of those SNPs in that HCV genotype.

## 2. Materials and Methods

### 2.1. Study Population

Two hundred and fifty-nine HCV-infected patients with genotype 1, with absence of coinfection by HIV or HBV and without chronic kidney disease, were recruited from Clementino Fraga Filho University Hospital (HUCCF) of Federal University of Rio de Janeiro, Brazil, between 2012 and 2015. All patients were at least 18 years old. All samples were previously genotyped by real time PCR in the hospital and had HCV viral strains belonging to genotype 1. Patients with previous treatment failure with pegIFN/RBV were classified according to the outcome into nonresponders or relapsers, whereas those with SVR after the treatment were classified as responders. All patients recruited received pegINF/RBV for 48 weeks, and there were no cases of patients who discontinued the therapy. Medical records provided by the hospital were consulted to access data of treatment response and viral genotype and subtype. The group of patients who successfully responded to the anti-HCV dual therapy was used as control to this study and was compared to the other groups for all five SNPs comprising the* IL28B* and the* IL10* genes. Considering a minor allele frequency (MAF) of 0.2 and the characteristics observed in comparisons between relapsers and responders (*N* = 61, fraction of cases = 0.57 and outcome prevalence = 0.28), which were the most stringent conditions observed in our study, the minimal OR values to reach a power of 80% were between 3.5 and 4.

In addition, a group of 112 healthy individuals was also included in the study as a population control, to determine allele frequencies and possible deviations from Hardy-Weinberg Equilibrium. All individuals had absence of coinfection by HIV, HBV, or HCV and were at least 18 years old. The present study has been approved by the Ethics Committee in Research of HUCCF with the number (CAAE) 22786113.8.0000.5257.

### 2.2. DNA Extraction, PCR, and Sequencing

Genomic DNA extraction were performed from blood samples with the PureLink® Genomic DNA Mini Kit (Invitrogen, Carlsbad, USA) following the manufacturer's protocol. DNA quantification was carried out in a Nanovue Plus spectrophotometer (GE Healthcare, Little Chalfont). PCRs for the* IL28B* rs12979860 and rs8099917 SNP analysis fragments were performed as described by Moreira et al. [12] and Sharafi et al. [13], respectively. The PCR for the* IL10* rs1800896, rs1800871, and rs1800872 SNPs was performed in a single DNA fragment spanning all three SNPs. The PCR reaction used 1x PCR Rx buffer, 2 mM MgCl_2_, 0.25 mM dNTP, 0.5 pmol of each primer 5′-CTGGCTCCCCTTACCTTCTAC-3′ (forward) and 5′-CCTAGGTCACAGTGACGTGG-3′ (reverse), 1U Taq polymerase (Invitrogen), and 50–500 ng of genomic DNA in a total volume of 50 *μ*L. The reaction cycling was 95°C for 3 min, followed by 35 cycles with 95°C for 45 sec, 57°C for 40 sec, and 72°C for 1 min, and a final extension step of 72°C for 7 min.

PCR amplification of DNA fragments was confirmed by 1.5% agarose gel electrophoresis and samples were then purified using the HiYield Gel/PCR DNA Mini kit (Real Genomics, Miami, USA), following the manufacturer's instructions. DNA sequencing was carried out using the same primers of the PCRs and the Big Dye Terminator Cycle Sequencing kit v3.1 (Life Technologies), according to the manufacturer's instructions, in an Applied Biosystems 3130 Genetic Analyzer platform (Life Technologies). Sequences were analyzed to visually identify the SNPs with the BioEdit program.

### 2.3. Data Analysis

Statistical analyses were performed with R project v. 3.3.1 [14]. Frequencies of each genotype and allele were determined by direct counting. Deviations from Hardy-Weinberg Equilibrium were assessed using data from healthy controls using a *χ*^2^ test. Frequencies of each SNP were compared between the three different patient groups (responders, nonresponders, and relapsers) using unconditional logistic regression models. Allele frequencies were compared among responders and nonresponders, responders and relapsers, and relapsers and nonresponders for each SNP using *χ*^2^ test.

A codominant model was considered primarily and, afterwards, analysis under dominant and recessive models was also performed when appropriate. Multivariate models were applied to combine the effect of* IL28B* SNPs. Pairwise linkage disequilibrium patterns were determined using *r*^2^ statistics. Haplotype frequencies were estimated by maximum likelihood and compared between the groups using logistic regression models. *p* values below 0.05 were considered significant.

## 3. Results

Overall, 125 patients were included in this study, 64 of which (51%) were nonresponders, 35 (28%) were relapsers, and 26 (21%) were responders. Of the participants, 56 (45%) were infected with HCV subtype 1a, while 56 (45%) were infected with HCV subtype 1b and only 1 (1%) carried HCV subtype 1c. Besides that, 12 (9%) did not have the subtype determined in their medical records. In addition, 58 (46%) of the patients were women and the median age was 56 years (Table 1). Different treatment outcomes did not show variations among age, sex, and other characteristics.

In the population control, formed by samples of healthy individuals, all frequencies of the five SNPs did not show deviations from Hardy-Weinberg Equilibrium: frequencies for rs12979860 were 0.52 CC, 0.34 CT, and 0.14 TT (*p* = 0.059); 0.71 TT, 0.24 TG, and 0.05 for rs8099917 (*p* = 0.179); 0.37 CC, 0.50 CA, and 0.13 AA for rs1800872 (*p* = 0.682); 0.39 CC, 0.47 CT, and 0.14 TT for rs1800871 (*p* = 1); and 0.46 AA, 0.44 AG, and 0.10 GG for rs1800896 (*p* = 1).

**Figure 1:**
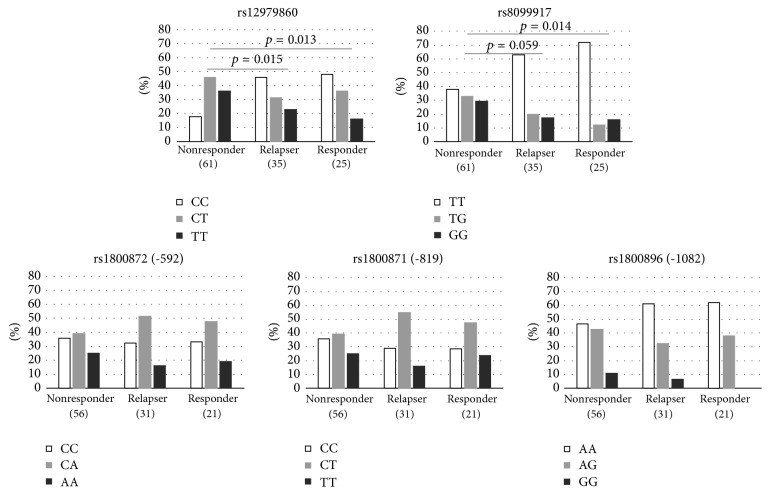
*IL28B* and* IL10* SNP frequency comparisons among nonresponders, relapsers, and responders. Frequencies were compared using chi-square test with 2 degrees of freedom. Significant values were found only for SNPs rs12979860 and rs8099917, both from the IL28B gene.

The frequency of the favorable genotype CC for the* IL28B* SNP rs12979860 was similar among responders and relapsers (0.48 and 0.46, respectively). As expected, the frequency of that genotype was significantly lower among nonresponders (0.18; *p* = 0.013 and 0.015 for comparisons with responders and relapsers, respectively) (Figure 1). The same trend was observed for the* IL28B* SNP rs8099917, with similar frequencies of genotype TT among responders and relapsers (0.72 and 0.63, respectively). Both patient groups were also different from nonresponders (*p* = 0.014 and 0.059, respectively), who showed a frequency of 0.38 for that genotype. No associations were observed for* IL10* SNPs rs1800896, rs1800871, and rs1800872 in the comparisons among responders, relapsers, and nonresponders frequencies.

Comparisons between responders and nonresponders confirmed the protective effect of rs12979860 CC (OR = 0.17; _95%_^ ^CI: 0.04–0.64; *p* = 0.009) and of rs8099917 TT (OR = 0.28; _95%_^ ^CI: 0.08–0.99; *p* = 0.047) in our population (Table 2). Results were similar when analysis was performed under a recessive model (OR = 0.24; *p* = 0.006 for rs12979860 CC versus CT/TT and OR = 0.23; *p* = 0.005 for rs8099917 TT versus GT/GG). Surprisingly, rs12979860 CC also showed a protective effect for relapsers compared to nonresponders in both codominant (OR = 0.25, _95%_^ ^CI: 0.08–0.76; *p* = 0.015) and recessive (0.26; 0.10–0.66; *p* = 0.005) models, as it occurs with responders (Table 3). No association was observed when frequency of* IL28B* SNPs was compared between relapsers and nonresponders (Table 3).

Haplotype analyses corroborated our findings, since the haplotype carrying allele C at rs12979860 and T at rs8099917 was associated with protection against the nonresponder phenotype when compared to responders (OR = 0.27; _95%_^ ^CI: 0.11–0.68; *p* = 0.007) or to relapsers (OR = 0.37; _95%_^ ^CI: 0.18–0.77; *p* = 0.009). Comparisons between responders and relapsers showed no difference between those groups, reinforcing that they are genetically similar (Table 4).

Multivariate analyses were also performed to assess a possible confounding effect between the* IL28B* SNPs in both codominant and recessive models. In comparisons between responders and nonresponders, the OR values obtained for rs12979860 CC remained similar after adjustment for rs8099917 in the codominant model (OR = 0.17; _95%_^ ^CI: 0.04–0.64 and _adj_^ ^OR = 0.18; _95%_^ ^CI: 0.04–0.76, respectively). When the recessive model was considered, the OR values were increased, suggesting that inclusion of rs8099917 has adjusted the effect of the rs12979860 CC genotype (OR = 0.24; _95%_^ ^CI: 0.09–0.66 and _adj_^ ^OR = 0.31; _95%_^ ^CI: 0.11–0.90, respectively). Comparisons between crude and multivariate models were statistically significant in both models (*p* = 0.04 and 0.02 for codominant and recessive models, respectively), suggesting that inclusion of rs8099917 reduced the model variance. However, association of both SNPs remained statistically significant in the multivariate model, suggesting an independent effect in this outcome (Table 2). Indeed, results of linkage disequilibrium analysis showed a low *r*^2^ (0.11) between* IL28B* SNPs in our population.

Again, no associations were observed for* IL10* promoter SNPs rs1800896, rs1800871, and rs1800872 in all analyses performed.

## 4. Discussion

This study describes that only* IL28B* SNPs showed significant differences in frequency between responders and nonresponders, as well as a protective effect of genotypes rs12979860 CC and rs8099917 TT against a nonresponder phenotype. The three* IL10* SNPs did not show differences between the compared groups. The latter SNPs are controversial in the literature, with some studies population showing an association with pegIFN/RBV therapy and others showing no effect [9, 15]. In this study, we did not find any significant association of* IL10* SNPs with response to therapy in a population of Rio de Janeiro, neither in frequency nor in OR. We have also compared all SNPs between nonresponders and relapsers in order to evaluate differences between those two types of patients. Once again, the* IL10* SNPs did not show any significant differences between the two groups, but* IL28B* SNPs rs12979860 and rs8099917 showed differences in frequency and also in the protective effect of genotypes CC and TT against being a nonresponder, just like the one seen between responders and nonresponders. Given that the union of both nonresponders and relapsers groups of therapy failure is common in the literature, our study highlights that they have significant differences in the frequency of* IL28B* SNPs rs12979860 and rs8099917, two of the most important SNPs associated with response to HCV therapy [11]. While nonresponders and responders had differences in the genotypic frequencies of rs12979860 and rs8099917, this did not occur when comparing relapsers and responders. Therefore, clustering nonresponders and relapsers can lead to an incorrect association or a lack of association between those SNPs and response to treatment. Responders and relapsers are similar on the distribution of the* IL28B* SNP genotypes. In fact, both groups have a good initial response to therapy since in both cases the HCV viral load gets undetectable, but at the end of the therapy responders remain undetectable while HCV rebounds in relapsers.

The* IL10* promoter SNPs rs1800872 (-592), rs1800871 (-819), and rs1800896 (-1082) did not show any association with any specific group of patients in this study, corroborating the controversial nature of the potential association of those SNPs with anti-HCV therapy, and in agreement with other reports that failed to observe significant associations [16, 17].

Until 2013, the mechanism of SNPs in the spontaneous cure of HCV was unclear. This fact changed with the discovery of the rs368234815 SNP very close to rs12979860, in the first exon of the new characterization human gene named IL-28C. This is a functional SNP, which the variation ΔG produces the interferon *λ*4 and the variation TT causes a change in the reading frame and culminates in a premature stop codon [18]. rs368234815 is now considered the true functional variant related to the treatment outcome: the production of interferon *λ*4 is unfavorable to hepatitis C virus clearance and it is associated with treatment failure [19]. This SNP is in strong linkage disequilibrium with rs12979860, with the allele TT equivalent to allele C and allele ΔG equivalent to allele T, respectively [18].

This study has a limitation that may be considered. pegIFN/RBV HCV treatment is no more the standard of care in HCV therapy after the arrival of DAA therapy in which the role of these polymorphisms is not as strong as in dual therapy. However, many underdeveloped countries still use pegIFN/RBV for treating HCV infection and thus these results might still have an impact on treatment outcome.

## 5. Conclusions

The frequency of* IL28B* SNPs rs12979860 and rs8099917 is different between relapsers and nonresponders, showing that those groups need to be analyzed separately in treatment efficacy comparisons. On the other hand, relapsers and responders are similar with respect to those* IL28B* SNP frequencies. In this study, the rs12979860 CC and rs8099917 TT genotypes, as well as the C/T haplotype, were associated with treatment success comparing nonresponse and response to the pegIFN/RBV therapy.

## Figures and Tables

**Table 1:** Demographic and clinical characteristics of patients infected with HCV genotype 1.

Variable	Total population (*n* = 125)
*Gender*	
Female (%)	58 (46%)
Male (%)	67 (54%)
*Age*	
Mean-median	54-56
*Viral load log10* ^*∗*^	
Mean-median	5.82-5.9
*AST/TGO*	
Mean-median	76.9 (66)
*Treatment outcome*	
Responders	26 (21%)
Relapsers	35 (28%)
Nonresponders	64 (51%)
*Genotype 1 diversity*	
Subtype 1a (%)	56 (45%)
Subtype 1b (%)	56 (45%)
Subtype 1c (%)	1 (1%)

^*∗*^Viral load before treatment.

**Table 2:** Association between *IL28B* and *IL10* SNPs and responders and nonresponders.

Genotype/haplotype	Responders	Nonresponders	OR (95% CI)	OR (95% CI)^*∗*^
rs12979860				
TT	4 (0.16)	22 (0.36)	Reference	Reference
CT	9 (0.36)	28 (0.46)	0.57 (0.15–2.08) (*p* = 0.392)	0.46 (0.12–1.80) (*p* = 0.268)
CC	12 (0.48)	11 (0.18)	0.17 (0.04–0.64) (**p** = 0.009)	0.18 (0.04–0.76) (**p** = 0.019)

CC versus CT/TT			0.24 (0.09–0.66) (**p** = 0.006)	0.31 (0.11–0.90) (**p** = 0.03)

rs8099917				
GG	4 (0.16)	18 (0.30)	Reference	Reference
GT	3 (0.12)	20 (0.33)	1.48 (0.29–7.54) (*p* = 0.636)	2.18 (0.40–11.93) (*p* = 0.369)
TT	18 (0.72)	23 (0.38)	0.28 (0.08–0.99) (**p** = 0.047)	0.42 (0.11–1.59) (*p* = 0.203)

TT versus GT/GG			0.23 (0.08–0.64) (**p** = 0.005)	0.29 (0.10–0.84) (**p** = 0.02)

rs1800872 (-592)				
CC	7 (0.33)	20 (0.36)	Reference	
CA	10 (0.48)	22 (0.39)	0.77 (0.25–2.41) (*p* = 0.65)	
AA	4 (0.19)	14 (0.25)	1.22 (0.30–4.99) (*p* = 0.77)	

CA/AA versus CC			0.9 (0.31–2.60) (*p* = 0.84)	

rs1800871 (-819)				
CC	6 (0.29)	20 (0.36)	Reference	
CT	10 (0.48)	22 (0.39)	0.66 (0.20–2.14) (*p* = 0.49)	
TT	5 (0.24)	14 (0.25)	0.84 (0.21–3.30) (*p* = 0.80)	

CT/TT versus CC			0.72 (0.24–2.14) (*p* = 0.56)	

rs1800896 (-1082)				
AA	13 (0.62)	26 (0.46)	Reference	
GA	8 (0.38)	24 (0.43)	0.67 (0.24–1.89) (*p* = 0.445)	
GG	0	6 (0.11)	not done	

GA/GG versus AA			1.88 (0.67–5.22) (*p* = 0.22)	

rs12979860/rs8099917				
T/G	0.13	0.31	Reference	
C/G	0.09	0.15	0.69 (0.16–3.03) (*p* = 0.623)	
C/T	0.57	0.26	0.27 (0.11–0.68) (**p** = 0.007)	
T/T	0.21	0.28	0.68 (0.24–1.88) (*p* = 0.455)	

OR: odds ratio; CI: confidence interval. ^*∗*^Multivariate models including rs12979860 and rs8099917. Comparisons between crude (rs12979860) and multivariate model including rs12979860 and rs8099917 were statistically significant in codominant (*p* = 0.04) and recessive models (*p* = 0.02). Haplotype frequencies were estimated by maximum likelihood.

**Table 3:** Association between *IL28B* and *IL10* SNPs and relapsers and nonresponders.

Genotype/haplotype	Relapsers	Nonresponders	OR (95% CI)
rs12979860			
TT	8 (0.23)	22 (0.36)	Reference
CT	11 (0.31)	28 (0.46)	0.93 (0.32–2.69) (*p* = 0.887)
CC	16 (0.46)	11 (0.18)	0.25 (0.08–0.76) (**p** = **0.015**)

CC versus CT/TT			0.26 (0.10–0.66) (**p** = **0.005**)

rs8099917			
GG	6 (0.17)	18 (0.30)	Reference
GT	7 (0.20)	20 (0.33)	0.95 (0.27–3.37) (*p* = 0.940)
TT	22 (0.63)	23 (0.38)	0.35 (0.12–1.04) (**p** = **0.059**)

TT versus GT/GG			0.36 (0.15–0.84) (**p** = 0.019)

rs1800872 (-592)			
CC	10 (0.32)	20 (0.36)	Reference
CA	16 (0.52)	22 (0.39)	0.69 (0.25–1.86) (*p* = 0.46)
AA	5 (0.16)	14 (0.25)	1.4 (0.39–4.99) (*p* = 0.60)

CA/AA versus CC			0.86 (0.34–2.17) (*p* = 0.74)

rs1800871 (-819)			
CC	9 (0.29)	20 (0.36)	Reference
CT	17 (0.55)	22 (0.39)	0.58 (0.21–1.59) (*p* = 0.29)
TT	5 (0.16)	14 (0.25)	1.26 (0.35–4.57) (*p* = 0.72)

CT/TT versus CC			0.74 (0.28–1.90) (*p* = 0.53)

rs1800896 (-1082)			
AA	19 (0.61)	26 (0.46)	Reference
GA	10 (0.32)	24 (0.43)	1.75 (0.68–4.51) (*p* = 0.24)
GG	2 (0.06)	6 (0.11)	2.19 (0.39–12.07) (*p* = 0.37)

GA/GG			1.83 (0.74–4.46) (*p* = 0.19)

rs12979860/rs8099917			
T/G	0.18	0.31	Reference
C/G	0.10	0.15	0.84 (0.28–2.54) (*p* = 0.752)
C/T	0.52	0.26	0.37 (0.18–0.77) (**p = 0.009**)
T/T	0.21	0.28	0.84 (0.36–1.93) (*p* = 0.675)

Haplotype frequencies were estimated by maximum likelihood. OR: odds ratio; CI: confidence interval.

**Table 4:** Association between *IL28B* and *IL10* SNPs and responders and relapsers.

Genotype/haplotype	Responders	Relapsers	OR (95% CI)
rs12979860			
TT	4 (0.16)	8 (0.23)	Reference
CT	9 (0.36)	11 (0.31)	0.61 (0.13–2.71) (*p* = 0.517)
CC	12 (0.48)	16 (0.46)	0.67 (0.16–2.74) (*p* = 0.574)

CC versus CT/TT			0.91 (0.32–2.55) (*p* = 0.86)

rs8099917			
GG	4 (0.16)	6 (0.17)	Reference
GT	3 (0.12)	7 (0.20)	1.56 (0.24–9.91) (*p* = 0.640)
TT	18 (0.72)	22 (0.63)	0.81 (0.20–3.34) (*p* = 0.776)

TT versus GT/GG			0.66 (0.22–2.00) (*p* = 0.46)

rs1800872 (-592)			
CC	7 (0.33)	10 (0.32)	Reference
CA	10 (0.48)	16 (0.52)	1.12 (0.32–3.90) (*p* = 0.86)
AA	4 (0.19)	5 (0.16)	0.87 (0.17–4.47) (*p* = 0.87)

CA/AA versus CC			1.05 (0.32–3.41) (*p* = 0.93)

rs1800871 (-819)			
CC	6 (0.29)	9 (0.29)	Reference
CT	10 (0.48)	17 (0.55)	1.13 (0.31–4.13) (*p* = 0.85)
TT	5 (0.24)	5 (0.16)	0.67 (0.13–3.34) (*p* = 0.62)

CT/TT versus CC			0.98 (0.29–3.32) (*p* = 0.97)

rs1800896 (-1082)			
AA	13 (0.62)	19 (0.61)	Reference
GA	8 (0.38)	10 (0.32)	0.85 (0.26–2.74) (*p* = 0.79)
GG	0	2 (0.06)	Not done

GA/GG versus AA			1.02 (0.32–3.20) (*p* = 0.96)

rs12979860/rs8099917			
T/G	0.13	0.18	Reference
C/G	0.09	0.10	0.83 (0.18–3.88) (*p* = 0.810)
C/T	0.57	0.52	0.76 (0.30–1.94) (*p* = 0.567)
T/T	0.21	0.21	0.82 (0.27–2.54) (*p* = 0.736)

Haplotype frequencies were estimated by maximum likelihood. OR: odds ratio; CI: confidence interval.

## Data Availability

The data used to support the findings of this study are available from the corresponding author upon request.

## References

[1] WHO (2016). *Bulletin of the World Health Organization - Fact sheet No. 164*.

[2] Messina J. P., Humphreys I., Flaxman A. (2015). Global distribution and prevalence of hepatitis C virus genotypes. *Hepatology*.

[3] Manns M. P., McHutchison J. G., Gordon S. C. (2001). Peginterferon alfa-2b plus ribavirin compared with interferon alfa-2b plus ribavirin for initial treatment of chronic hepatitis C: a randomised trial. *The Lancet*.

[4] Fields B. N., Knipe D. M., Howley P. M. (2013). *Fields Virology*.

[5] Ge D., Fellay J., Thompson A. J. (2009). Genetic variation in IL28B predicts hepatitis C treatment-induced viral clearance. *Nature*.

[6] Suppiah V., Moldovan M., Ahlenstiel G. (2009). IL28B is associated with response to chronic hepatitis C interferon-*α* and ribavirin therapy. *Nature Genetics*.

[7] Afzal M. S., Tahir S., Salman A. (2011). Analysis of interleukin-10 gene polymorphisms and hepatitis C susceptibility in Pakistan. *The Journal of Infection in Developing Countries*.

[8] Edwards-Smith C. J., Jonsson J. R., Purdie D. M., Bansal A., Shorthouse C., Powell E. E. (1999). Interleukin-10 promoter polymorphism predicts initial response of chronic hepatitis C to interferon alfa. *Hepatology*.

[9] Dogra G., Chakravarti A., Kar P., Chawla Y. K. (2011). Polymorphism of tumor necrosis factor-*α* and interleukin-10 gene promoter region in chronic hepatitis C virus patients and their effect on pegylated interferon-*α* therapy response. *Human Immunology*.

[10] TarragÔ A. M., da Costa A. G., Pimentel J. P. D. (2014). Combined impact of hepatitis C virus genotype 1 and interleukin-6 and tumor necrosis factor-*α* polymorphisms on serum levels of pro-inflammatory cytokines in Brazilian HCV-infected patients. *Human Immunology*.

[11] Eslam M., Leung R., Romero-Gomez M. (2014). IFNL3 polymorphisms predict response to therapy in chronic hepatitis C genotype 2/3 infection. *Journal of Hepatology*.

[12] Moreira S., Garcia R. F. L., Gutberlet A. (2012). A straightforward genotyping of the relevant IL28B SNPs for the prediction of hepatitis C treatment outcome. *Journal of Virological Methods*.

[13] Sharafi H., Pouryasin A., Alavian S. M. (2012). Distribution of IL28B genotypes in Iranian patients with chronic hepatitis C and healthy individuals. *Hepatitis Monthly*.

[14] Core Team R. (2015). *R: A language and environment for statistical computing. R Foundation for Statistical Computing*.

[15] Umemura T., Joshita S., Yoneda S. (2011). Serum interleukin (IL)-10 and IL-12 levels and IL28B gene polymorphisms: Pretreatment prediction of treatment failure in chronic hepatitis C. *Antiviral Therapy*.

[16] Abdelraheem W. M., Hassuna N. A., Abuloyoun S. M., Abdel Ghany H. M., Rizk H. A., Abdelwahab S. F. (2016). Interleukin-10.rs1800896 and Interleukin-18.rs1946518 gene polymorphisms could not predict the outcome of hepatitis C virus infection in Egyptian patients treated with pegylated interferon plus ribavirin. *Archives of Virology*.

[17] Constantini P. K., Wawrzynowicz-Syczewska M., Clare M. (2002). Interleukin-1, interleukin-10 and tumour necrosis factor-alpha gene polymorphisms in hepatitis C virus infection: An investigation of the relationships with spontaneous viral clearance and response to alpha-interferon therapy. *Journal of Liver*.

[18] Prokunina-Olsson L., Muchmore B., Tang W. (2013). A variant upstream of *IFNL3* (*IL28B*) creating a new interferon gene *IFNL4* is associated with impaired clearance of hepatitis C virus. *Nature Genetics*.

[19] O'Brien T. R., Prokunina-Olsson L., Donnelly R. P. (2014). IFN-*λ*4: the paradoxical new member of the interferon lambda family. *Journal of Interferon & Cytokine Research*.

